# Promoter Binding by the Adr1 Transcriptional Activator May Be Regulated by Phosphorylation in the DNA-Binding Region

**DOI:** 10.1371/journal.pone.0003213

**Published:** 2008-09-15

**Authors:** Nataly Kacherovsky, Christine Tachibana, Emily Amos, David Fox, Elton T. Young

**Affiliations:** Department of Biochemistry, University of Washington, Seattle, Washington, United States of America; East Carolina University, United States of America

## Abstract

**Background:**

Post-translational modification regulates promoter-binding by Adr1, a Zn-finger transcriptional activator of glucose-regulated genes. Support for this model includes the activation of an Adr1-dependent gene in the absence of Adr1 protein synthesis, and a requirement for the kinase Snf1 for Adr1 DNA-binding. A fusion protein with the Adr1 DNA-binding domain and a heterologous activation domain is glucose-regulated, suggesting that the DNA binding region is the target of regulation.

**Methodology/Principal Findings:**

Peptide mapping identified serine 98 adjacent to the Zn-fingers as a phosphorylation site. An antibody specific for phosphorylated serine 98 on Adr1 showed that the level of phosphorylated Adr1 relative to the level of total Adr1 decreased with glucose derepression, in a Snf1-dependent manner. Relative phosphorylation decreased in a *PHO85* mutant, and this mutant constitutively expressed an Adr1-dependent reporter. Pho85 did not phosphorylate Adr1 *in vitro*, suggesting that it affects Adr1 indirectly. Mutation of serine 98 to the phosphomimetic amino acid aspartate reduced *in vitro* DNA-binding of the recombinant Adr1 DNA-binding domain. Mutation to aspartate or alanine affected activation of a reporter by full-length Adr1, and *in vivo* promoter binding.

**Conclusions/Significance:**

Mutation of Adr1 serine 98 affects *in vitro* and *in vivo* DNA binding, and phosphorylation of serine 98 *in vivo* correlates with glucose availability, suggesting that Adr1 promoter-binding is regulated in part by serine 98 phosphorylation.

## Introduction

Glucose repression ensures that yeast cells use preferred carbon sources until available supplies are exhausted. Much of the regulation of glucose-repressed genes occurs at the level of transcription [1], [2], and in yeast, Adr1 has a pivotal role in expressing these genes [3], [4]. Control of Adr1 occurs at several levels: through transcription of its gene [5]; post-translational modification of the protein [6]; and access to, or ability to stably bind promoters [7], [8]. The latter two mechanisms appear to be the most critical, because in glucose-repressed cells, raising Adr1 protein levels to be comparable to those in derepressed cells does not activate transcription of *ADH2* or other Adr1-regulated genes [6], [9]. Adr1 does not bind its cognate promoters in these conditions, so even at elevated protein levels, post-translational and promoter-binding regulation keep its activity in check [6], [7].

Several lines of evidence suggest regulation of Adr1 binding and activity by post-translational modification. Derepression of the Adr1-regulated gene *ADH2* can occur in the absence of protein synthesis [6]. Snf1, a central kinase in transcription of glucose-regulated genes, is required for Adr1 promoter-binding [7]. Snf1 is the yeast homolog of the AMP-activated protein kinase, and activation of Snf1 in repressing conditions relieves some glucose repression of *ADH2* and other Adr1-dependent genes, allowing up to 10% of derepressed transcription levels [8], [10]. Snf1 does not appear, however, to phosphorylate and activate Adr1 directly, as Adr1 lacks a consensus site for Snf1 phosphorylation and no Snf1-Adr1 *in vitro* phosphorylation or association has been detected (N. Kacherovsky, K. Dombek, unpublished). Since Snf1 is not required for *ADR1* expression [5] but is required for DNA-binding by Adr1, one hypothesis is that Snf1 indirectly regulates Adr1 post-translationally. Protein kinase A (PKA) has been implicated in direct phosphorylation of Adr1. Both yeast and mammalian PKA phosphorylate Adr1 *in vitro* [11], and inactivation of *BCY1*, encoding the regulatory subunit of protein kinase A, inhibits Adr1 expression and activity [10], [12]. Whether PKA phosphorylates Adr1 *in vivo* has not been resolved, however [13]. Adr1 has a PKA consensus site at Ser230, and mutations of this residue or in this region lead to alleles called *ADR1^c^* that allow constitutive *ADH2* expression and hyper-derepression [11], [12]. Nonetheless, the hyper-active Adr1*^c^* proteins are still sensitive to glucose repression and respond to activation of Snf1 in repressing conditions, pointing to regulation beyond the potential Ser230 modification [8], [10].

Another possible site for post-translational control of Adr1 is its DNA binding domain (DBD, residues 76–165). Fusion of the Adr1 DBD to a heterologous activation domain confers glucose-regulated expression on target promoters. Conversely, a Gal4 DBD - Adr1 (amino acids 21–1323) fusion shows no glucose regulation of Gal4 targets [6]. The Adr1 DBD contains two canonical C2H2 zinc fingers (amino acids 99–160) and an amino terminal region of approximately 20 amino acids called PAR (proximal accessory region) that is essential for high affinity binding [14], [15]. The structure of the DBD has been determined by NMR in the presence and absence of DNA. The accessory motif PAR is unstructured in the absence of DNA and assumes a more ordered conformation when bound to DNA [16]. Mutations in PAR affect DNA binding: changing Pro 87 or Pro 97 to Ala or Gly abrogates DNA binding and activity *in vivo* [17], while mutation of Arg 91 to Lys allows better *in vitro* binding and suppresses several loss-of-function mutations in the DBD [18], [19].

Because phosphorylation is a common mechanism of post-translational modification, and the Adr1 DBD appears to be crucial to glucose repression, we looked for phosphorylation of the Adr1 DBD as a possible mode of regulation. Serine 98, which is in a region critical for PAR structural changes during DNA-binding [16], appeared to be phosphorylated *in vitro* and *in vivo*. Mutation of serine 98 affected DNA-binding and activation of Adr1-dependent genes, suggesting that it could play a role in glucose repression. A screen of yeast kinase deletion mutants identified the kinase complex Pho80/85 as affecting the phosphorylation state of Adr1 serine 98 and contributing to glucose repression of some Adr1-dependent genes.

## Results

### The Adr1 DBD is phosphorylated *in vitro* and *in vivo*

**Figure 1:**
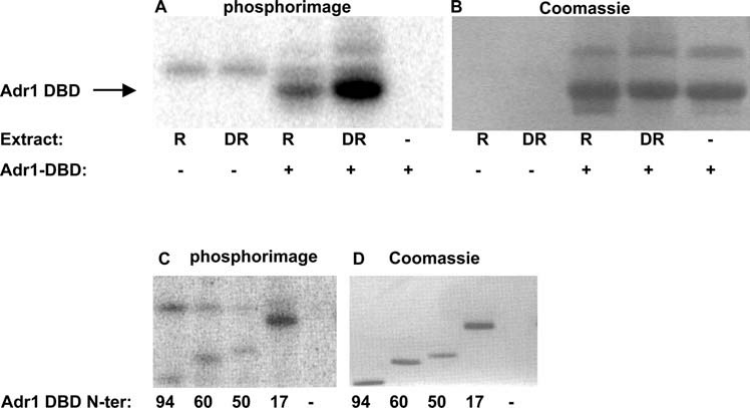
Phosphorylation of Adr1 DBD by cell extracts. A. *In vitro* kinase assay with purified, recombinant Adr1 DBD (amino acids 17–160 of Adr1), [γ-^32^P]-ATP and cell extracts from TYY201. R, cells were grown in repressing medium; DR, cells were derepressed in 0.05% glucose for 3 hours. Because of variability in the extracts, quantitative comparisons cannot be made between the repressed and derepressed lanes. B. Coomassie stain of the gel in A. C. *In vitro* kinase assay as in A, but with N-terminally truncated versions of the Adr1 DBD. Numbers indicate the amino acid in wild-type Adr1 that is the N-terminus of the truncated version. D. Coomassie stain of the gel in C.

Binding of DNA by Adr1 is a regulated step [7] and the DBD of Adr1 contains a number of serine and threonine residues that could be phosphorylated. To assess whether the Adr1 DBD might be a target for a yeast protein kinase, we incubated recombinant Adr1 DBD with yeast whole cell extracts and [γ-32P]-ATP. SDS gel electrophoresis followed by autoradiography showed a prominent band at the position of the Adr1 DBD (18 kDa) that was absent if recombinant protein was omitted from the mixture (Figure 1A, B and Table 1 for yeast strains). N-terminal truncations of the Adr1 DBD showed that the site of phosphorylation was within amino acids 94–160, which includes the C-terminal portion of the proximal accessory region (PAR) and the Zn-fingers (Figure 1C, D). Two-dimensional peptide mapping and amino acid analysis of tryptic peptides from the phosphorylated protein (data not shown) suggested a single major site of phosphorylation at the serine within the sequence RTPSGK at amino acids 95–100.

**Table 1:** Yeast strains

strain	genotype	source
BY4741 and kinase deletions	*MATa his3*Δ*1 leu2*Δ*0 met15*Δ*0 ura3*Δ*0* and relevant gene knockout with *kanMX*	[43]
CKY13	*MATa ade2-1 can1-100 ura3-1 leu2-3,112 trp1-1 his3-11,115* Δ*adr1::kanMX*	this work
VBY20	*MATα adh3 ura3 his3 leu2::*(pRS315*-ADR1*)X3 *ADH2::*YIp*ADH2/lacZ*(*TRP1*) *ADH2::*YIp*ADH2/GFP*(*URA3*) *ADR1*-3HA::kanmx	[9]
NK85	VBY20 *ADR1*-3HA::kanmx	this work
NK87	NK85 Δ*snf1*::*kanmx*	this work
NK135	NK85 Δ*pho85*::*NAT1*	this work
NK136	NK85 Δ*pho80*::*NAT1*	this work
NK139	NK85 Δ*pcl10*::*NAT*	this work
TYY201	*MATa ade2 can1-100 his3-11,115 leu2-3,112 trp1-1 ura3-1*	[32]
TYY497	*MATa ade2 can1-100 his3-11,115 leu2-3,112 trp1-1 ura3-1* Δ*adr1*::*LEU2 ADH2*::YIp*ADH2/lacZ*(*trp1*::*HIS3*)	[32]

**Figure 2:**
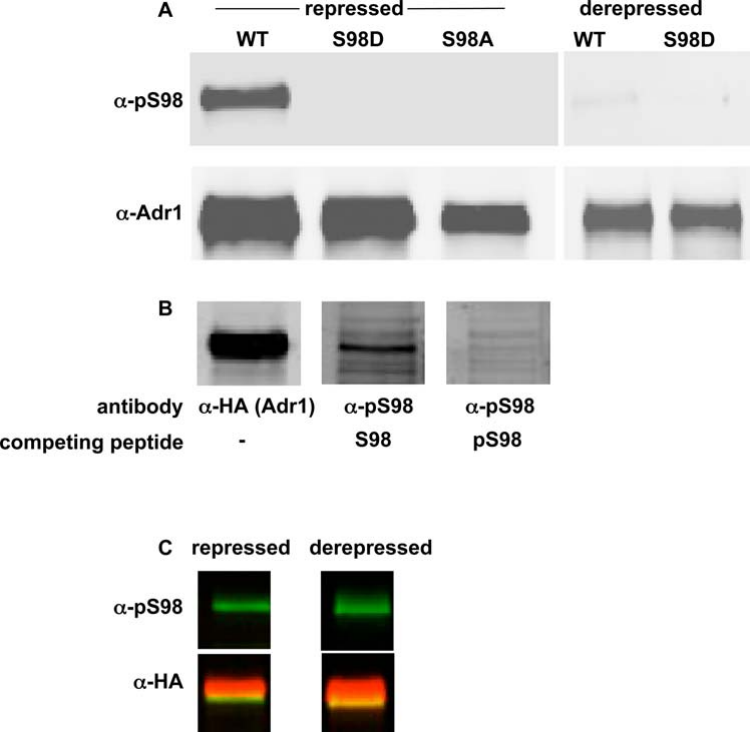
*In vivo* phosphorylation of Adr1 at serine 98. A. Western blots with antibody against phosphorylated serine 98 of Adr1 (α-pS98) or total Adr1 (α-Adr1 or α-HA) were performed on 50 µg of cell extract per lane from Δ*adr1* strain TYY497 with high-copy plasmid pNKA1 (WT, wildtype Adr1), pNKA2 (Adr1-S98D) or pNKA4 (Adr1-S98A). Derepressed cells were grown in 0.05% glucose for one hour. B. Western blots were performed as in A with α-pS98 or α-HA epitope antibody. Each lane contains 20 µg of repressed cell extract from TYY497 with plasmid pKD17-HA. Competing phosphorylated (pS98)-, or non-phosphorylated Ser98 (S98)-containing peptides were added to the primary antibody at 10 µg/ml. C. Western blots were performed as in B on 50 µg per lane of NK91. Derepressed cells were grown in 0.05% glucose, 2% ethanol for one hour. In this section, the lower panel shows the merged images of α-pS98 and α-HA.

To determine if serine 98 was phosphorylated *in vivo*, we used antibodies raised against LRLNGRTP(pS)GKLRSFVC, where (pS) is phosphorylated serine. Adr1 is difficult to detect in repressed conditions, so we used strains containing a multi-copy plasmid with *ADR1* under control of the constitutive *ADH1* promoter. To study the specificity of the antiserum and the effect of point mutants that mimic the non-phosphorylated and phosphorylated state, serine 98 on Adr1 was changed to alanine and aspartate in related plasmids, referred to as S98A and S98D. The anti-phosphoserine 98 peptide antiserum (α-pS98) recognized a protein in yeast cell extracts that co-migrated with Adr1, was absent in cells lacking Adr1 (data not shown), and was greatly reduced when Adr1-S98A or Adr1-S98D was expressed instead of wild-type Adr1 (Figure 2A). The specificity of α-pS98 was confirmed when the phosphorylated peptide efficiently competed with the protein detected on the western blot, but a non-phosphorylated synonymous peptide did not (Figure 2B). In summary, the serine 98 proximal to the Zn-finger domain of Adr1 is phosphorylated both *in vitro* and *in vivo*.

### Adr1-Ser98 phosphorylation is affected by glucose repression and *SNF1*

If phosphorylation of Adr1 at serine 98 is relevant to its activity *in vivo*, we would expect the level of phosphorylation to change in glucose derepressing conditions. We measured serine 98 phosphorylation in extracts from cells shifted to low glucose by western blotting with anti-pS98 and anti-Adr1 antiserum. The intensity of the anti-pS98 signal of each sample was normalized to its signal with anti-Adr1. We found that derepression was accompanied by a decrease in serine 98 phosphorylation relative to total Adr1 of five-fold (average of three separate experiments, standard deviation of two, Figure 2A). Glucose regulation of Adr1 occurs through *SNF1* because Adr1 does not bind promoters in a Δ*snf1* strain [7]. To test the effect of Snf1 on Adr1 serine 98 phosphorylation, we used strains with four integrated copies of *ADR1* expressed from its own promoter, one of which was tagged with a triple HA epitope. Glucose repression is maintained in this strain despite a modestly elevated level of Adr1 [6]. Figure 2C shows Adr1 serine 98 phosphorylation in a strain lacking Snf1. The decrease in phosphorylation relative to total Adr1 in low glucose was less than two-fold (average of two experiments, standard deviation of 0.4), so *SNF1* was required for the full decrease in serine 98 phosphorylation normally seen in low glucose.

### Adr1-serine98 mutants have reduced DNA binding affinity *in vitro* and *in vivo*

**Figure 3:**
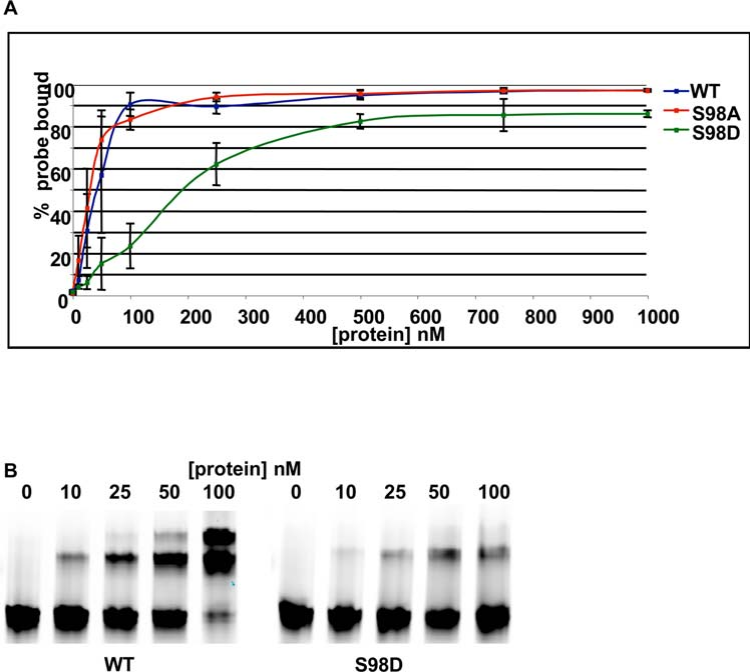
*In vitro* binding of Adr1DBD and Ser98 mutants. A. Electrophoretic mobility shift assays (EMSA) were performed with increasing amounts of Adr1 DBD (amino acids 17–160) and its Ser98Ala (S98A) and Ser98Asp (S98D) variants, against a fluorescently labeled double-stranded oligonucleotide containing the Adr1 binding site (UAS1) from the *ADH2* promoter. Blue line, wildtype; red line S98A; green line S98D. Error bars represent the standard deviation of the mean measured in duplicate experiments. B. EMSA gel showing mobility shifts of the *ADH2* promoter probe with wildtype Adr1 DBD or the S98D mutant.

Serine 98 phosphorylation decreases in low glucose, while Adr1 promoter-binding increases, and both processes are Snf1-dependent ([7], Figure 2). Therefore, we hypothesized that the DNA-binding of Adr1 would be affected by its serine 98 phosphorylation state. In support of this hypothesis, serine 98 is in a region previously shown to be important for DNA binding, the PAR adjacent to the Zn-finger domain [15], [16], [20]. The DNA binding affinity for recombinant proteins from the wild-type, S98A and S98D plasmids were measured using an electrophoretic mobility shift assay (EMSA). Figure 3 shows that the neutral S98A mutation did not affect *in vitro* DNA binding, but the phosphomimetic S98D mutation reduced DNA binding affinity approximately 10-fold. The apparent equilibrium constant of Adr1-S98D was 200nM compared to about 20nM for the wild-type and S98A mutant proteins. Other mutations in PAR, such as at proline 97, similarly reduced DNA binding affinity *in vitro* [20].

**Figure 4:**
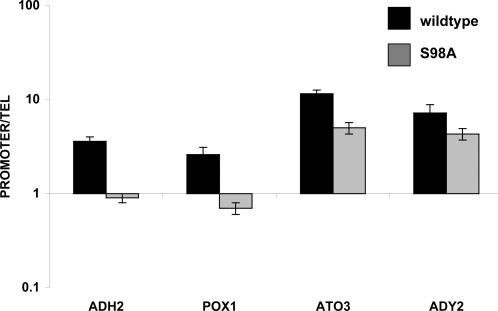
*In vivo* binding of Adr1 and S98A mutant. Chromatin immunoprecipitation assays were performed using anti-HA antibody on the Δ*adr1* strain TYY497 with pKD16-HA (wildtype, HA-tagged Adr1, black bars) and pKD16-9-HA (Ser98Ala, HA-tagged Adr1, gray bars) derepressed in 0.05% glucose for 3 hours. Quantitation was performed by RTqPCR for the indicated promoters normalized to a non-Adr1-bound telomeric region. Error bars are the standard deviation for the average of technical replicates.

Chromatin immunoprecipitation (ChIP) analysis with quantitation by real-time PCR (qPCR) was used to compare *in vivo* promoter-binding of wild-type Adr1 and the S98 mutants. As expected, binding of the Adr1-S98D mutant was at or below background levels, even when over-expressed from a 2μ plasmid under *ADH1* promoter-control (data not shown). The Adr1-S98A mutant, however, gave unexpected results. When expressed from a CEN plasmid to ensure physiological levels, binding of the S98A mutant was reduced strongly at *ADH2* compared to wildtype Adr1 (Figure 4). Binding was also assayed at *POX1*, *ATO3* and *ADY2*, which are all highly glucose-repressed, strongly Adr1-dependent for expression, and have promoters that are bound by Adr1. *ADH2*, *ADY2* and *ATO3* are Snf1-dependent, but *POX1* is not [4], [21]. Binding of the S98A mutant was as strongly affected at *POX1* as it was at *ADH2*, and was significantly decreased at *ATO3*. In summary, mutation of Adr1 serine 98 to the phosphomimetic amino acid aspartate reduced DNA binding affinity *in vitro* and led to loss of *in vivo* binding detection. Mutation to alanine did not affect *in vitro* DNA binding, but reduced *in vivo* binding.

### Mutation of Adr1-Ser98 to Asp or Ala decreases transcription activation

Since mutation of serine 98 affected the ability of Adr1 to bind promoters, we tested the effect on activation of glucose-repressed genes. First, a yeast strain lacking Adr1 was transformed with multi-copy plasmids expressing wild-type Adr1, Adr1-S98A or Adr1-S98D under the control of the *ADH1* promoter. The activity of over-expressed Adr1 was assayed using an Adr1-responsive integrated *ADH2*/lacZ reporter gene. Wild-type Adr1 was active in both repressing and derepressing medium as expected, because over-expression of Adr1 to this level overcomes glucose repression ([22], [23], Table 2). Despite the high level of expression, the S98D mutant had very low activity in both repressing and derepressing conditions. The S98A mutant was slightly less active than the wild-type protein. Western blots for Adr1-HA protein showed similar levels of wild-type and mutant proteins, indicating that the inactivity of Adr1-S98D was not due to poor expression or instability (data not shown).

**Table 2:** Beta-galactosidase assays on *ADH2* promoter-*lacZ* with high-copy *ADR1* wildtype or S98 mutants

strain a	ß-galactosidase activity (Miller Units)	
Adr1	R^b^	DR
WT	340 (50)^c^	630 (170)
S98A	260 (47)	290 (100)
S98D	6 (0.1)	30 (3.0)

a TYY497 with pNKA1, pNKA2 or pNKA4

b R, repressed; DR, derepressed 0.05% glucose 2% ethanol, 2 hours

c Numbers in parentheses are standard deviations for the average of three independent cultures

**Figure 5:**
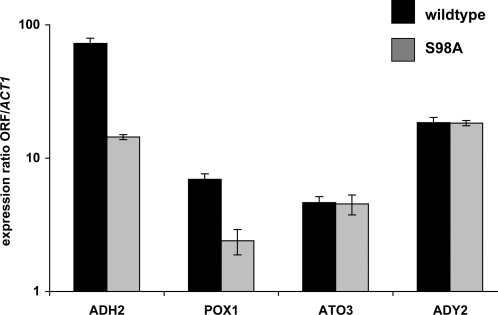
Activation of Adr1-dependent genes by Adr1 or the S98A mutant. RTqPCR on RNA isolated from Δ*adr1* strain CKY13 with pKD16-HA (wildtype, HA-tagged Adr1, black bars) and pKD16-HA-9 (S98A, HA-tagged Adr1, gray bars) derepressed in 0.05% glucose for 3 hours. Quantitation was for the indicated genes normalized to *ACT1*. Error bars are the standard deviation for the average of technical replicates.

Some *ADH2* expression was seen in a strain with the Adr1-S98A mutant, in contrast to the Adr1-S98D mutant strain, which had less than 10% of wildtype expression levels. To examine the Adr1-S98A mutant at physiological levels, the S98A mutant allele was transferred to a CEN plasmid and expressed under the control of its own promoter. The defective transcription activation of this mutant was confirmed and extended to other Adr1-dependent genes by measuring RNA levels with quantitative real-time PCR (Figure 5). *ADH2* expression was reduced 5-fold. Expression of the Adr1-dependent gene *POX1* was reduced 2.9-fold. Two other Adr1-dependent genes, *ATO3* and *ADY2* were activated equally well by mutant and wild-type Adr1, consistent with the higher level of *in vivo* Adr1-binding at these promoters (Figure 4).

### Pho85 is involved in Adr1-Ser98 phosphorylation

**Figure 6:**
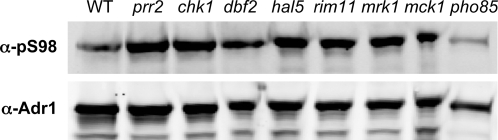
Effect of Δ*pho85* on Adr1 phosphorylation. Western blots using antibody against phosphorylated serine 98 of Adr1 (α-pS98) or total Adr1 (α-Adr1) were performed on 99–125 µg of cell extract per lane from BY4741 wildtype or strains with the indicated kinase gene deletion. Each strain carried the high-copy Adr1 plasmid pNKA1-U.

Binding and gene expression data showed that serine 98, proximal to the Zn-finger domains, is important for the function of Adr1. Since its phosphorylation state correlated with glucose availability, we identified potential kinases by screening for Adr1-phosphoserine 98 in a collection of 102 yeast strains, each deleted for a gene encoding a non-essential kinase. Each kinase mutant was transformed with a multi-copy plasmid expressing Adr1 from the *ADH1* promoter. Phosphorylation of serine 98 was measured and normalized to total Adr1 using immunoprecipitation with anti-Adr1 antiserum followed by western blotting with either anti-pS98 or anti-Adr1 antiserum. Figure 6 shows the screening of a subset of mutants by western blot. For the wild-type strain, the signal ratio for the two antibodies was 0.17 (+/− 0.04, for three independent cultures). Three kinase mutations showed altered levels of serine 98 phosphorylation: *pho85* (signal ratio 0.08), *dun1* (0.27), and *ssk2* (0.11). We focused on *pho85* since it has been reported to have a role in growth control at the diauxic transition [24]. Dun1 is important for DNA damage control [25] and Ssk2 is involved in responding to osmotic stress [26]. Adr1 has not been implicated in either of these processes.

Pho85 is a cyclin-dependent protein kinase with multiple cyclin partners [27]. Yeast strains with deletions in genes encoding Pho85-associated cyclins were taken from the yeast deletion collection and transformed with the multi-copy *ADH1* promoter-*ADR1* plasmid. Screening by ADHII activity assays [28] and Western blotting as described above, indicated that Pho80 might affect Adr1 phosphorylation and Adr1-dependent gene expression (data not shown). We then tested the effects of deleting the genes for the *PHO85* kinase, its cyclin partner *PHO80* and two cyclins that had been negative in the initial screen, on transcription activation by Adr1. Strains containing an *ADH2* promoter/*lacZ* reporter and four integrated copies of *ADR1* [6], were deleted for *PHO85* or Pho85-associated cyclin genes. *PHO85* and *PHO80*, but not *PCL6* or *PCL10* affected *ADH2*-*lacZ* expression by beta-galactosidase assays in repressed conditions and blue/white color on X-gal-glucose plates (Table 3 and data not shown).

**Table 3:** Beta-galactosidase assays with *ADH2* promoter-lacZ in Δ*pho80*, Δ*pho85*, Δ*pcl10*

strain a	ß-galactosidase activity (Miller Units)
WT	5.3 (1.7)^b^
Δ*pho85*	180 (103)
Δ*pho80*	28 (1.1)
Δ*pcl10*	2.6 (0.8)

a NK85, NK135, NK136 or NK139 grown in YPD 5% glucose

b Numbers in parentheses are standard deviations for the average of duplicate cultures

To test for direct phosphorylation of the Adr1-DBD by Pho80/85, *in vitro* kinase assays [29] were performed with purified Pho80/85 complex (a gift from Ian Carter-O'Connell from Erin O'Shea's lab) on purified recombinant Adr1 DBD, or the unphosphorylated 17-amino acid serine 98-containing peptide that was generated for affinity purification of α-pS98. We could not detect phosphorylation of the Adr1 DBD with the purified kinase-cyclin. We assume the effect of *PHO85* on Adr1 phosphorylation that is seen in Figure 6 is indirect, since purified Pho80/85 could phosphorylate a purified Pho4 control, but did not phosphorylate the Adr1 DBD or the serine 98-containing peptide (data not shown).

## Discussion

### Serine 98 of Adr1 is involved in regulation of DNA-binding

Adr1 directly regulates at least 30 glucose-repressed genes [4], [21] and is itself regulated at the level of DNA-binding [7]. We found that mutation of serine 98 in the PAR region of Adr1, which is crucial for optimal binding [15], [16], [20], [30], had a severe effect on *in vivo* binding to, and activation of the *ADH2* promoter. Mutation to the phosphomimetic amino acid aspartate had more deleterious effects than mutation to alanine on both *in vitro* binding and activation, suggesting that phosphorylation of serine 98 might have similar consequences. The effect of the alanine mutation on Adr1-dependent genes ranged from 5-fold or greater decreases in binding and activation (*ADH2*), to no effect (*ADY2*). This may reflect differences in the structure and coactivator requirements of the different promoters, which we find leads to measurable differences in the DNA binding or activator function of Adr1 [31]. Serine 98 is adjacent to the proline at position 97 that creates a turn in the structure of the DBD [16], which may explain why even mutation to a neutral amino acid affects activator function. Serine 98 was phosphorylated *in vivo*, and phosphorylation was affected by both glucose availability and Snf1. *SNF1* was required for the decrease in phosphorylation in response to derepressing conditions.

**Figure 7:**
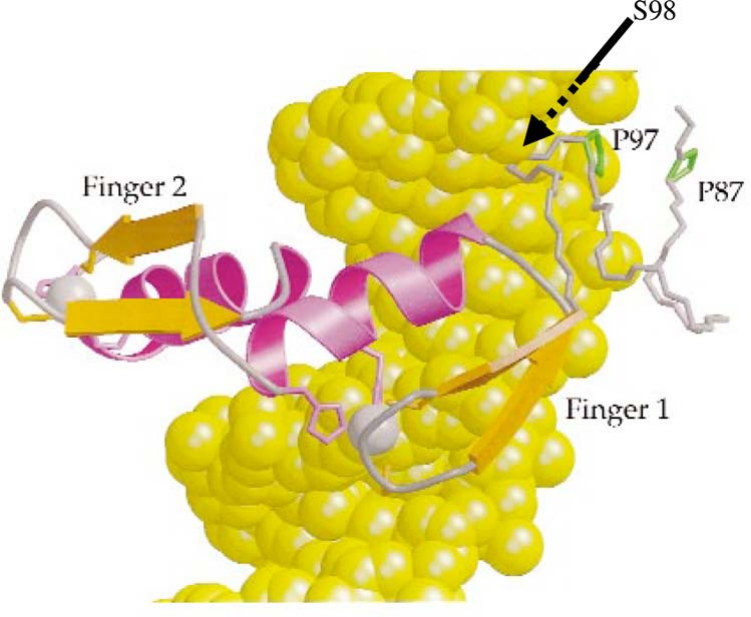
Model of the Adr1 DBD - DNA interaction, showing the position of serine 98. A model of the Adr1 PAR and DBD (ribbon structure) bound to its recognition sequence in DNA (space-filling model), based on NMR spectra of the complex in solution. Fingers 1 and 2 are the Zn-fingers; P87 and P97 are prolines; the arrow points to serine 98. Reprinted by permission from Macmillan Publishers Ltd: Nat Struct Biol 6:478–485, copyright 1999.

Several possible mechanisms could explain how post-translational modification of Adr1 may regulate promoter binding and transcription activation. Adr1 promoter-binding may be stabilized by interactions with coactivators [32], and post-translational modification may regulate this interaction. A plausible hypothesis is that phosphorylation interferes with coactivator interaction. However, the close proximity of serine 98 to the DBD of Adr1 (Figure 7) decreases the likelihood of direct coactivator binding. In addition, the Adr1 DBD binds DNA in absence of coactivators in the *in vitro* EMSA assay, suggesting a reasonably stable binding without assistance. Another candidate for regulating interaction with stabilizing coactivators is serine 230 on Adr1. This residue also appears to be phosphorylated, with relative levels of phosphorylation changing in derepression (S. Ratnakumar and N. Kacherovsky, unpublished). Since this residue is far from the DNA-binding domain, it is a better candidate for regulating interactions with coactivators. Since serine 98 is in a region known to be important for DNA-binding, and mutation affects binding *in vivo* and *in vitro*, we propose that phosphorylation of this residue directly affects Adr1-DNA interactions. A model of the Adr1 DBD binding to DNA based on nuclear magnetic resonance spectroscopy of the complex in solution, shows the serine 98 in a position to influence interactions between the PAR domain and DNA. (Figure 7). Since mutation of serine 98 affects binding *in vivo* and *in vitro*, our current working hypothesis is that phosphorylation of this residue affects Adr1-DNA interactions.

*In vivo* evidence suggested the Pho80/85 kinase/cyclin complex contributed to phosphorylation of Adr1 at serine 98; however, *in vitro* evidence suggested the effect was indirect, and serine 98 is not within a Pho85 consensus sequence [33]. We had observed that the Δ*pho85* strain with the high-copy *ADR1* plasmid grew approximately 75% slower than a *PHO85* strain carrying the same plasmid, suggesting a physiologically relevant interaction between Pho85 and Adr1. The slower growth rate was partially because of the *pho85* mutation, but the growth rate of the Δ*pho85* strain with the high-copy *ADR1*-S98D plasmid was only 19% slower than the comparable *PHO85* strain. The synthetic deleterious effect between high-copy *ADR1* and Δ*pho85*, with partial rescue by mutation of the phosphorylatable residue on Adr1 suggests a common pathway. Pho85 involvement in Adr1 regulation was also observed in a gene expression microarray performed in a Δ*pho85* strain undergoing the diauxic transition [24]. Many Adr1-dependent genes were characterized as having elevated expression in the *pho85* mutant before the cells had exhausted their supply of glucose. Pho85, paired with the cyclin subunits Pcl8 and Pcl10, is involved in glycogen synthesis, in the same pathway as Snf1 [34], and a *pho85* mutant has growth defects on non-glucose carbon sources [35]. Here we present data that support a function for Pho85 in glucose repression.

## Materials and Methods

### Strains and plasmids

Yeast strains are listed in Table 1. Epitope-tagging was as described in Knop et al. [36] and gene deletion was as described in Güldener et al. [37].

Cells were grown as described in Sherman [38]. For glucose repression, medium contained 5% glucose; for derepression, 0.05% glucose with or without 2% ethanol.

Plasmid pF1F2 (Adr1 17–160 a.a.) [15] was used to express the Adr1 DBD in *E. coli* for purification. Plasmids F1F2S98A and F1F2S98D were generated from pF1F2 using the Stratagene QuickChange kit. pNKA1, pNKA2, pNKA4, which have the genes for wildtype, S98D and S98A Adr1 DBD, respectively, were made by GAP repair [39] using PCR fragments generated from pF1F2, pF1F2S98D, and pF1F2S98A, cotransformed into yeast with linearized pKD46, which has *ADR1* under control of the *ADH1* promoter, on a 2μ-TRP1 plasmid (K. Dombek, unpublished)**.** pNKA1-U is pNKA1 with the *TRP1* marker swapped for *URA3* according to Cross [40]. pKD16-HA and pKD17-HA are pKD16 and pKD17 [5] tagged as described in Knop et al. [36]. The Serine 98 in pKD16-HA-9 was mutated to alanine using the QuickChange kit (Stratagene).

### *In vitro* phosphorylation of Adr1

Purification of Adr1 DBD (F1F2) or the S98A or S98D variants from *E. coli* was as described in Young et al. [4] from pF1F2, F1F2S98A or F1F2S98D. For *in vitro* phosphorylation of the Adr1 DBD, 1–10 µg of Adr1F1F2 was bound to Ni-NTA magnetic agarose beads (Qiagen) through its His6 tag, and resuspended in kinase buffer (10mM Tris pH 7.5, 10mM MgCl2, 20mM beta-mercaptoethanol or 10mM dithiothreitol, 0.005% Tween 20, 10mM imidazole, 2mM ATP, 1–5 µCi per reaction of 3000Ci/mmol [γ-^32^P]-ATP). An equal volume of cell extract made according to Thukral et al. [30] was added, and after 15–60 minutes at 37°C, the reaction was stopped by washing the beads four times in 10mM Tris pH 8.0, 300mM NaCl, 10mM imidazole, 0.005% Tween 20. Adr1 DBD was eluted by boiling in 4× LDS loading buffer (Invitrogen), run on a 12% gel (Invitrogen) with protein standards, and visualized with a Storm Phosphorimager. Yeast protease inhibitor cocktail (Sigma), 1mM PMSF, and phosphatase inhibitors (1 mM sodium pyrophosphate, 1 mM sodium orthovanadate, 1 mM ß-glycerophosphate, 1 mM EGTA and 10 mM sodium fluoride), were added to extracts and wash buffers.

Tryptic peptide mapping and phosphoamino acid analysis were performed as described in Boyle et al. [41] using Adr1 DBD phosphorylated as for the *in vitro* phosphorylation by cell extracts. The Pepsort program http://pingu.salk.edu/sefton/Hyper_protocols/pepsort1.html generated the graph with predicted tryptic peptide electrophoretic patterns.

### *In vivo* phosphorylation of Adr1

Gel electrophoresis and Western blots were carried out on yeast extracts generated according to Horvath and Reizman [42] and Hahn, S “Rapid yeast protein prep for SDS PAGE and Western,” http://www.fhcrc.org/science/labs/hahn/methods/biochem_meth/yeast_prot_SDS.html with addition of a 30 second bead-beating step before boiling. Tris-Acetate 3–8% gels were run, Western blotted, visualized and quantitated according to manufacturer's instructions for the NuPage system (Invitrogen) and Odyssey Infrared imaging system (Licor Biosciences). Polyclonal primary antibodies were used at 1∶500–1∶1000 and were anti-HA (Y-11, Santa Cruz Biochemicals), anti-Adr1 [10] or antibodies against phosphoserine 98 of Adr1. For the latter, the peptide LRLNGRTP(pS) GKLRSFVC and its unphosphorylated version were synthesized and used to generate and affinity purify the anti-pS98 antibody by Bethyl Laboratories (Montgomery, TX). Secondary IR-dye conjugated antibodies used at 1∶1000–1∶3000 were goat anti-mouse Alexa 680 (Molecular Probes) or IRdye800 conjugated anti-rabbit IgG (Rockland Immunochemicals).

The screen for yeast mutants that affected Adr1 phosphorylation used a subset of 102 strains from the *MATa* yeast gene deletion collection (Invitrogen) that encode protein kinases according to the Saccharomyces Genome Database (yeastgenome.org). Strains were transformed with pNKA1-U. After growth under glucose repressing conditions, cell extracts were made by bead beating in lysis buffer (50mM Hepes pH 7.5, 140 mM NaCl, 1% Triton X-100, 0.1% NaDeoxycholate, with protease and phosphatase inhibitors as described above). After centrifugation, protein concentration was determined with the Bio-Rad protein assay kit, and 1 mg of soluble extract was incubated with 5–10 µl anti-Adr1 antibody [10] for 3 hours at 4°C. After centrifugation, the supernatant was transferred to a tube with 60 µl of a 1∶1 suspension of protein A sepharose : PBS (GE Healthcare) and incubated on a rocking platform for 1 hour, 4°C. After 3 washes with lysis buffer, Adr1 was eluted with 60 µl 4× LDS sample buffer (Invitrogen) at 70°C for 10 minutes and analyzed by Western blot with as described above.

### Expression of Adr1-dependent genes and Adr1 DNA binding

RNA expression and *ADH2* promoter - *lacZ* reporter activity was measured as described in Tachibana et al. [8]. ChIP assays were performed as described in Young et al. [4]. The sequences of oligonucleotides used for cloning and diagnostics are available on request.

### Electrophoretic Mobility Shift Assays (EMSA)

EMSA was conducted as in Thukral et al. [17], using a double-stranded probe containing the UAS1 sequence (underlined) from the *ADH2* promoter (sequence of top strand: 5′ GCA TTG ACT AAG TTC TCC AAC TTA TAA GTT GGA GAT GAA TCA GTT ACG 3′) labeled with an infrared dye and quantitated with an Odyssey Infrared Imaging System (Li-Cor Biosciences). Signals from all shifted bands in a lane were divided by the combined signal for all bands in the lane to determine % bound.
